# Reduced IQGAP2 promotes esophageal squamous cell carcinoma by regulating MEK/ERK MAPK pathway

**DOI:** 10.3389/fgene.2026.1815586

**Published:** 2026-08-10

**Authors:** Liangcheng Zhao

**Affiliations:** Department of Gastroenterology, The Fifth People’s Hospital of Wujiang District, Suzhou, Jiangsu, China

**Keywords:** cell proliferation, ESCC, *in vitro*, IQGAP2, MEK/ERK MAPK

## Abstract

**Background:**

IQ motif-containing GTPase activating protein 2 (IQGAP2) has been identified as a tumor suppressor in various cancers; however, its role in esophageal squamous cell carcinoma (ESCC) remains unclear.

**Methods:**

We analyzed IQGAP2 expression in tumor tissues from 236 ESCC patients using RNA sequencing and validated the findings with qPCR. EC9706 ESCC cell lines with IQGAP2 overexpression and knockdown were established via lentiviral transfection and selection. Cell proliferation was assessed using the CCK-8 assay, and activation of the MEK/ERK MAPK pathway was evaluated by Western blotting.

**Results:**

IQGAP2 expression was significantly decreased in tumor samples from ESCC patients. Overexpression of IQGAP2 markedly inhibited proliferation of ES9706 cells, whereas knockdown of IQGAP2 promoted cell proliferation. Mechanistically, decreased IQGAP2 expression enhances ESCC cell proliferation by activating the MEK/ERK MAPK signaling pathway.

**Conclusion:**

Our findings highlight IQGAP2 as a potential therapeutic target in ESCC, warranting further investigation for the development of effective treatments.

## Introduction

Esophageal squamous cell carcinoma (ESCC) is a common and aggressive malignancy with a rising global incidence, responsible for approximately 544,100 deaths in 2020, thereby posing a significant challenge in oncology (Morgan et al., 2022). The disease is often diagnosed at an advanced stage, limiting therapeutic options and contributing to poor patient prognosis (Lin et al., 2014; Abnet et al., 2018). Although various treatments-including surgery, chemotherapy, and radiotherapy-have been employed, overall survival rates remain unsatisfactory (Nakajima and Kato, 2013; Liao et al., 2019; Doki et al., 2022). This highlights the urgent need to elucidate the molecular mechanisms underlying ESCC pathogenesis to facilitate the development of more effective therapeutic strategies.

The IQ motif-containing GTPase activating protein (IQGAP) family has drawn attention for its complex roles in tumorigenesis (White et al., 2009; Shannon, 2012; Morgan et al., 2019; Wei and Lambert, 2021). Among its members, IQGAP2 is recognized as a key tumor suppressor, involved in regulating cell proliferation, migration, and adhesion (Xie et al., 2019; Xu et al., 2020; Kumar et al., 2021; Song et al., 2023). Several studies have demonstrated that IQGAP2 inhibits cancer cell growth and metastasis through modulation of diverse signaling pathways (Xu et al., 2020; Kumar et al., 2022; Song et al., 2023). Of particular interest is its role in regulating the MEK/ERK mitogen-activated protein kinase (MAPK) pathway, a critical axis in cell growth and survival (Kumar et al., 2021). Although the precise molecular interplay between IQGAP2 and the MEK/ERK MAPK pathway remains under investigation, emerging evidence emphasizes IQGAP2’s importance in maintaining cellular homeostasis and inhibiting tumorigenesis across multiple cancer types (Kumar et al., 2021; Wang et al., 2021; Kumar et al., 2022).

To date, the role of IQGAP2 in ESCC has not been explored. Given its potential therapeutic relevance, this study aims to investigate how reduced IQGAP2 expression influences the MEK/ERK MAPK signaling pathway and promotes ESCC cell proliferation. The findings may offer valuable insights into the molecular pathogenesis of ESCC and suggest IQGAP2 as a potential target for novel therapeutic interventions.

## Materials and methods

### Patients

Tissue samples were obtained from 236 patients diagnosed with ESCC who underwent esophagectomy at our hospital between 2017 and 2019. The collected specimens included formalin-fixed, paraffin-embedded (FFPE) tumor tissues and matched adjacent non-tumorous tissues located more than 5 cm from the tumor margins. All samples were histopathologically confirmed by experienced pathologists. None of the patients received radiotherapy or chemotherapy prior to surgery. Tumor staging was conducted according to the American Joint Committee on Cancer (AJCC) eighth edition guidelines (Rice et al., 2017). Detailed clinical and pathological characteristics of the enrolled patients are presented in Table 1.

**TABLE 1:** Characteristics of the study populations.

Clinical characteristics	Cases	Percent
Age (years)		
≥64	148	62.7
<64	88	37.3
Gender		
Male	178	75.4
Female	58	24.6
T stage		
T1	23	9.7
T2	53	22.5
T3	147	62.3
T4	13	5.5
N stage		
N0	153	64.8
N1	48	20.3
N2	27	11.4
N3	8	3.4
M stage		
M0	225	95.3
M1	11	4.7
Clinical stage		
Locoregional (I–II)	47	19.9
Advanced (III–IV)	189	80.1

Ethical approval for this study was granted by the Ethics Committee of the Fifth People’s Hospital of Wujiang District, and written informed consent was obtained from all participants. RNA sequencing (RNA-seq) of the ESCC tissue samples was performed by BGI Genomics (Shenzhen, China).

### Transcriptome deep sequencing

Whole RNA-seq was performed on normal esophageal tissues and matched ESCC tumor tissues from three patients by BGI Genomics using the MGISEQ-2000 sequencing platform, as previously described (Lang et al., 2021). For visualization, a heatmap was generated using OmicShare tools, a freely accessible online data analysis platform (http://www.omicshare.com/tools). Gene Ontology (GO) analysis of the RNA-seq data was conducted using the R programming language.

### qPCR

Quantitative polymerase chain reaction (qPCR) assays were conducted using the TaqMan method, following the manufacturer’s protocol. Primers and TaqMan Gene Expression Assay probes for the target genes were obtained from Thermo Fisher Scientific Inc. The assays were performed in duplicate, including a no-template negative control to ensure specificity. qPCR reactions were carried out on the CFX96 Touch Real-Time PCR Detection System (Bio-Rad, Hercules, USA). The specific probes used in this study were c-Myc, Cyclin D1, MMP9, MEK, ERK, IQGAP2, and GAPDH, with GAPDH serving as the internal control. Relative gene expression levels were calculated using the 2^−ΔΔCT^ method.

### Cell culture

EC9706, a poorly differentiated ESCC cell line, was obtained from the Chinese Academy of Medical Sciences (Beijing, China). The cells were cultured in RPMI-1640 medium supplemented with 10% fetal bovine serum, 100  U/mL penicillin, and 100 μg/mL streptomycin (Thermo Fisher Scientific Inc., Waltham, USA). Cultures were maintained in a humidified incubator with 5% CO_2_ at 37 °C.

### Plasmid construction and lentiviral transfection

The IQGAP2 overexpression construct (pCDH-MSCV-MCS-IQGAP2) and the short hairpin RNA (shRNA) construct targeting IQGAP2 (pHY-LV-KD-IQGAP2) were synthesized and obtained from Sangon Biotech (Shanghai, China). Lentiviral packaging and production were carried out by Genomeditech (Shanghai, China). EC9706 cells were transduced with either the IQGAP2 overexpression lentivirus (IQGAP2-OE) or the IQGAP2 knockdown lentivirus (shIQGAP2). Untreated parental cells were used as the control for IQGAP2 overexpression experiments and knockdown experiments. Following infection, cells were subjected to puromycin selection to generate stable 1QGAP2-overexpressing and IQGAP2-knockdown cell lines.

### Western blot

EC9706 cells were lysed using RIPA buffer supplemented with a 1× protease inhibitor cocktail (Thermo Fisher Scientific, Waltham, USA) and 1% phenylmethylsulfonyl fluoride (Sigma-Aldrich, St. Louis, USA) to preserve protein integrity. Protein concentrations were determined using the BCA Protein Assay Kit (Beyotime, Shanghai, China). Equal amounts of total protein (20 μg per lane) were separated by SDS-polyacrylamide gel electrophoresis (Thermo Fisher Scientific) and subsequently transferred onto polyvinylidene fluoride membranes (Millipore, Burlington, USA).

Membranes were blocked with 5% non-fat milk and incubated overnight at 4 °C with the following primary antibodies: anti-IQGAP2 (#MA5-44712, Thermo Fisher, 1:1,500), anti-MEK (#9122, CST, 1:1,000), anti-phospho-MEK (Ser217/221, #9154, CST, 1:1,000), anti-ERK (#4695, CST, 1:2000), anti-phospho-ERK (Thr202/Tyr204, #4370, CST, 1:1,000), and anti-GAPDH (#2118, CST, 1:2000). After washing, membranes were incubated with horseradish peroxidase-conjugated secondary antibodies, and bands were visualized using enhanced chemiluminescence (Sigma-Aldrich). Band intensity was quantified using ImageJ software.

### CCK-8 assay

To assess the proliferative capacity of EC9706 cells with IQGAP2 overexpression or knockdown, cells were seeded into 96-well plates at a density of 1 × 10^3^ cells per well. Cell viability was measured at 0, 24, 48, and 72 h using the Cell Counting Kit-8 (CCK-8; Beyotime). At each time point, 10 μL of CCK-8 reagent was added to each well and incubated for the recommended duration. Absorbance was then measured at 450 nm using a microplate reader, serving as a quantitative indicator of cell viability. The 0-h reading, obtained after cells adhered to the plate, served as the baseline. MEK-specific inhibitor rescue experiment was conducted to confirm pathway dependence. We performed a gold-standard rescue experiment using the MEK-specific inhibitor PD98059 (10 μM) in IQGAP2-knockdown EC9706 cells, with three groups set as follows: shControl + DMSO, shIQGAP2 + DMSO, shIQGAP2 + PD98059.

### Statistical analysis

All statistical analyses were performed using GraphPad Prism 9.0 (https://www.graphpad.com/). Continuous variables were expressed as mean ± standard deviation (SD). Comparisons between groups were conducted using Student’s t-test (two-tailed, unpaired) or two-way ANOVA, as appropriate. P-values less than 0.05 were considered statistically significant. In this study, GO enrichment analysis was performed using the clusterProfiler R package (version 4.8.3), and the org. Hs.e.g.,.db R package (version 3.18.0) was used for human gene ID conversion.

## Results

### The expression of IQGAP2 in ESCC samples of patients

**FIGURE 1:**
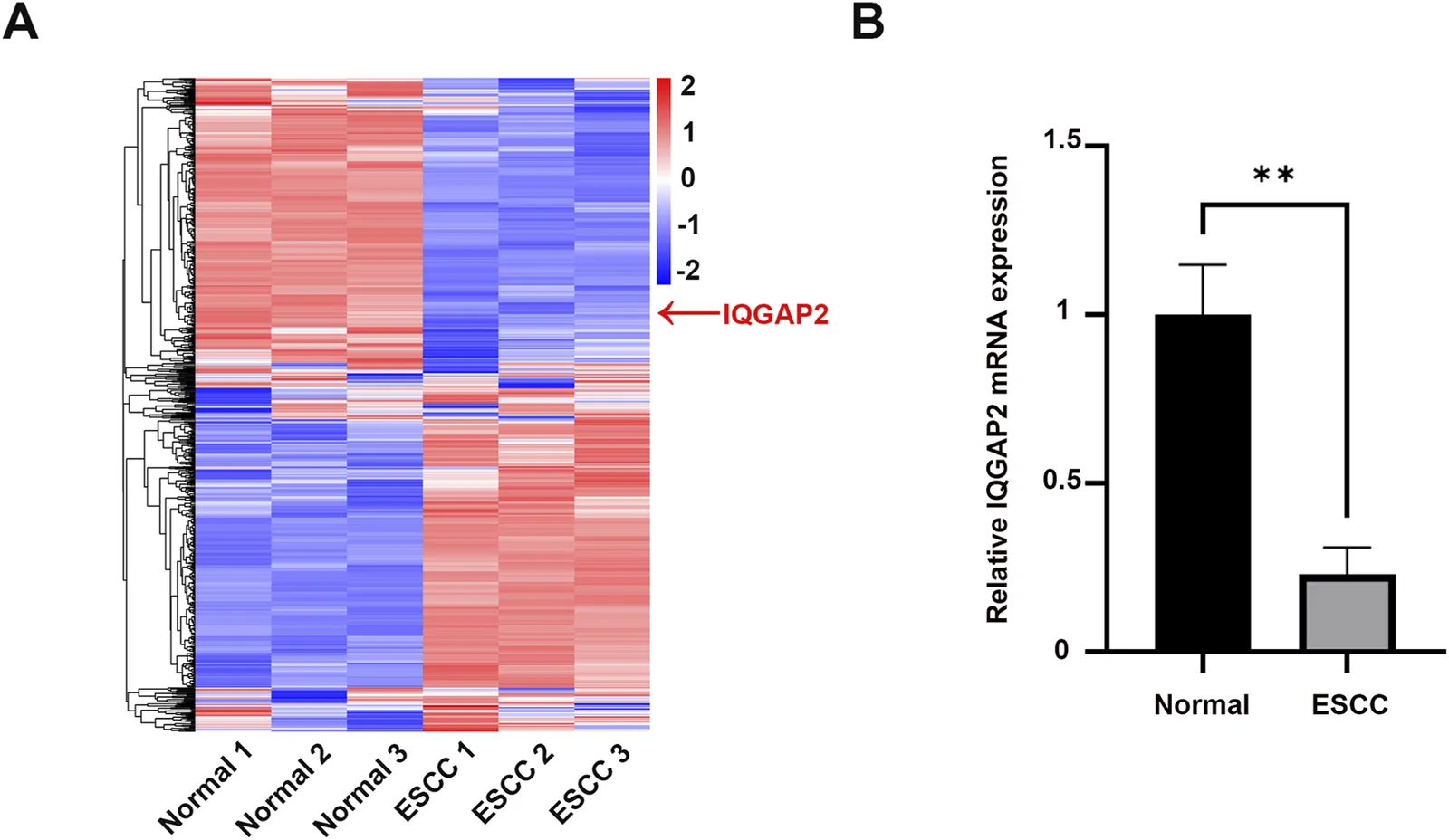
Expressions of IQGAP2 in ESCC samples and normal tissue. **(A)** Heatmaps. Red represents high levels of expression and purple represents low levels of expression. N = 3 patients. **(B)** The expression levels of IQGAP2 in the ESCC samples and normal tissue were measured by qPCR. Data are represented as mean ± SD. N = 236 patients. T-test was performed; **p < 0.01.

A total of 236 patients diagnosed with ESCC were enrolled in this study, and the clinical characteristics of the cohort are summarized in Table 1. RNA-seq analysis revealed a set of differentially expressed genes between tumor and adjacent normal tissues. Among them, IQGAP2 was notably downregulated in ESCC tumor tissues, as illustrated by the heatmap in Figure 1A. To validate these findings, qPCR was performed on all 236 paired tissue samples. Consistently, qPCR results confirmed a significant decrease in IQGAP2 expression in tumor tissues compared to adjacent non-tumorous tissues (Figure 1B).

To analyze the correlation between IQGAP2 expression and clinicopathological parameters, we divided the 236 patients into IQGAP2 high-expression and low-expression groups based on the median IQGAP2 expression level, and performed Pearson’s chi-square test to analyze the correlation between IQGAP2 expression and clinicopathological parameters (Supplementary Table S1). The results showed that low IQGAP2 expression was significantly associated with higher T stage (*p* = 0.002), lymph node metastasis (N+, *p* < 0.001), and advanced clinical stage (stage III-IV, P < 0.001), while no significant correlation was found with age or gender. These results directly demonstrate that downregulation of IQGAP2 is closely related to the malignant progression of ESCC, supporting its role as a biomarker for the malignant phenotype of ESCC. According to the Kaplan-Meier analysis, the ESCC patients who presented with low IQGAP2 expression had a shorter survival time than the patients with high IQGAP2 expression (Supplementary Figure S1). Furthermore, we investigated the functional pathways of IQGAP2 through GSEA. The enrichment outcomes revealed a significant accumulation of IQGAP2 in the MAPK signaling pathway (Supplementary Figure S2). The results showed that low expression of IQGAP2 was accompanied by exceptional activation of several important cancer-related pathways, of which the MAPK signaling pathway was the most significant.

### Effects of IQGAP2 on ESCC cell proliferation *in vitro*

**FIGURE 2:**
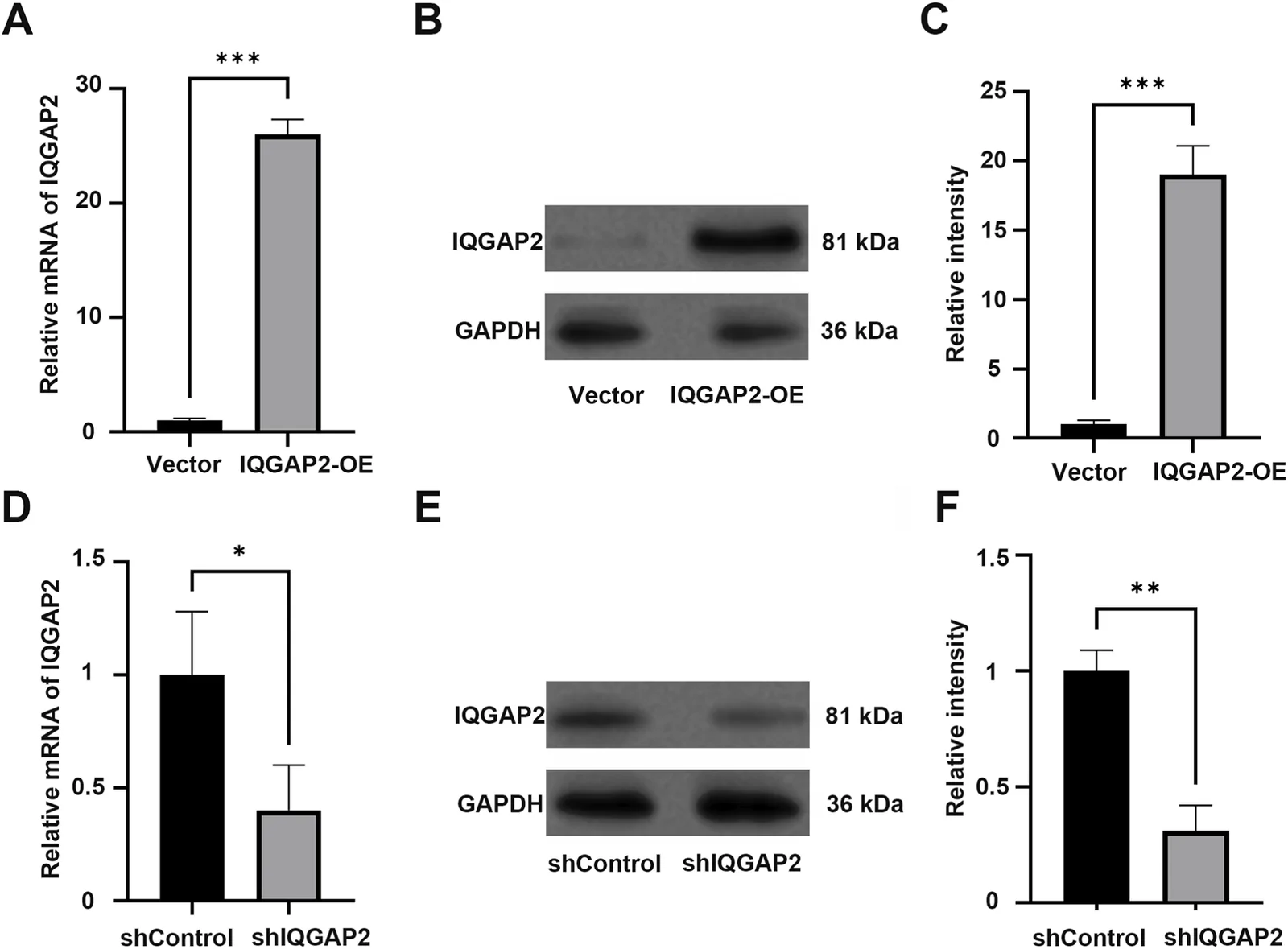
Overexpression or knockdown of IQGAP2 in EC9706 cells. The expression levels of IQGAP2 in the EC9706 were measured by qPCR **(A,D)** and Western blotting **(B,E)**. The quantified relative band intensity was determined by ImageJ **(C,F)**. Overexpression: IQGAP2-OE, knockdown: shIQGAP2. Data are represented as mean ± SD. N = 3. T-test was performed; *p < 0.05. **p < 0.01. ***p < 0.001.

To explore the role of IQGAP2 in ESCC progression, EC9706 cell lines with stable 1QGAP2 overexpression (IQGAP2-OE) and knockdown (siIQGAP2) were established. Both mRNA and protein levels of IQGAP2 were significantly increased in EC9706 cells transfected with the IQGAP2-OE plasmid, as demonstrated by qPCR and Western blot analyses (Figures 2A–C). Conversely, treatment with siIQGAP2 resulted in a marked reduction of IQGAP2 expression at both the mRNA and protein levels (Figures 2D–F).

**FIGURE 3:**
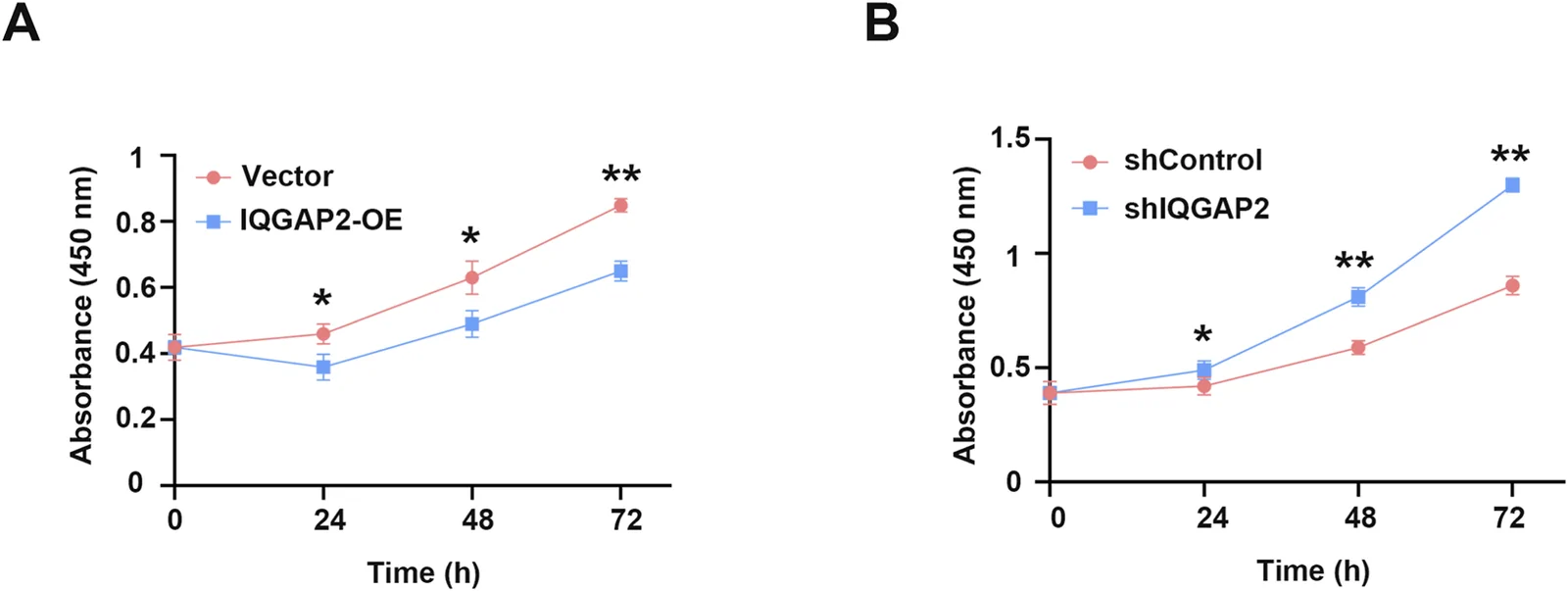
IQGAP2 inhibits ESCC cell proliferation *in vitro*. CCK-8 assays comparing cell growth of IQGAP2-OE **(A)** or shIQGAP2 **(B)** cells as compared with their controls. Data are represented as mean ± SD. N = 3. T-test was performed; *p < 0.05. **p < 0.01.

Functionally, overexpression of IQGAP2 significantly inhibited the proliferative capacity of EC9706 cells compared to controls (Figure 3A). In contrast, knockdown of IQGAP2 notably enhanced EC9706 cell proliferation (Figure 3B).

### IQGAP2 knockdown promotes ESCC cell proliferation through regulation of MEK/ERK MAPK pathway

**FIGURE 4:**
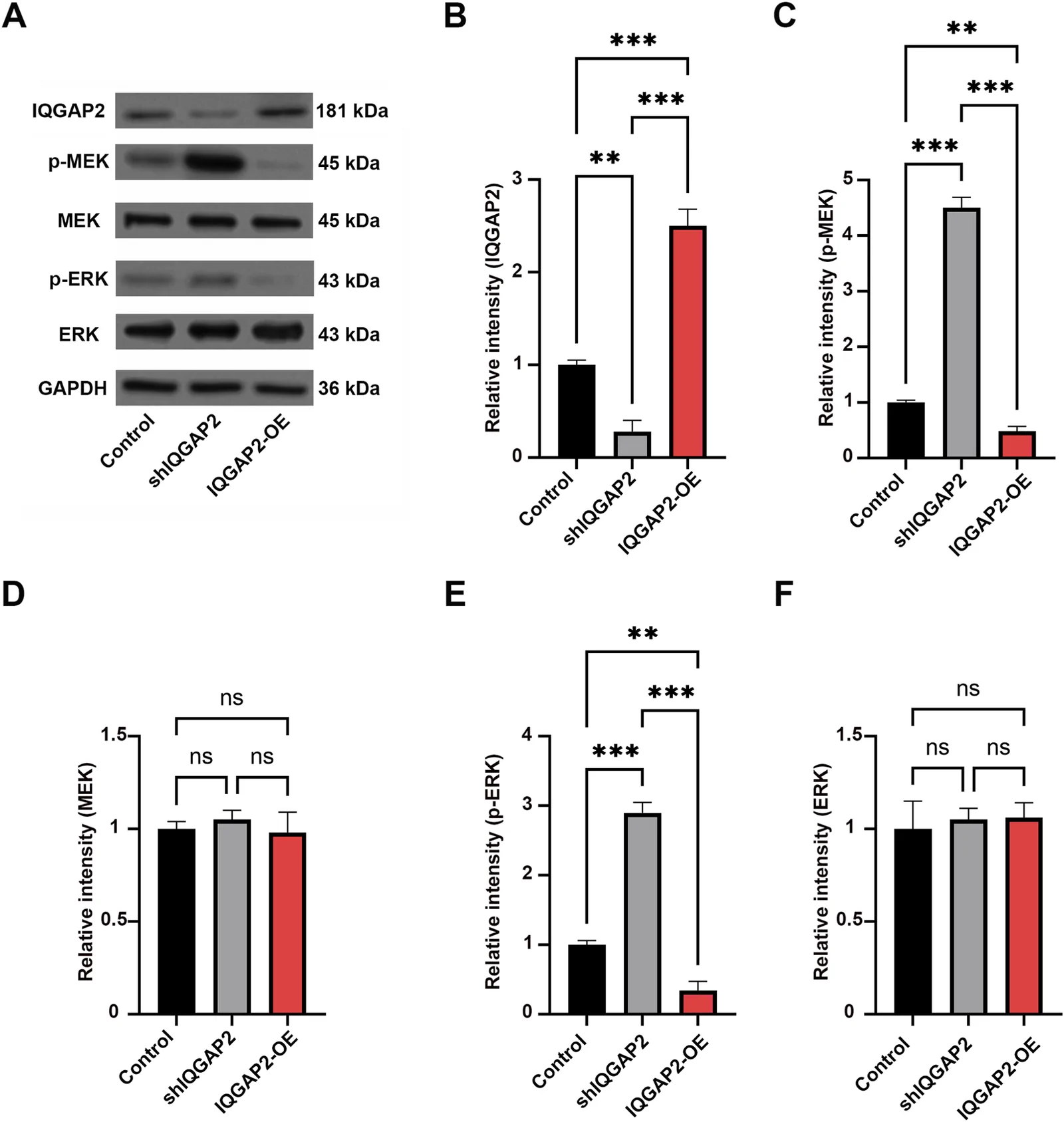
Reduced IQGAP2 promotes esophageal squamous cell carcinoma cell proliferation through regulation of MEK/ERK MAPK pathway. **(A)** Western blot showing the expression of phospho-MEK and phospho-ERK in EC9706 with IQGAP2 overexpression (IQGAP2-OE) or knockdown (shIQGAP2). GAPDH was used as a loading control. The quantified relative band intensity was determined by ImageJ **(B–F)**. Data are represented as mean ± SD. N = 3. ANOVA was performed; **p < 0.01; ***p < 0.001.

The MEK/ERK MAPK pathway plays a critical role in the survival and development of various tumor cells (Guo et al., 2020). To investigate whether IQGAP2 modulates this pathway in ESCC, changes in MEK/ERK signaling were analyzed in EC9706 cells following IQGAP2 overexpression or knockdown (Figures 4A,B). Western blot analysis demonstrated that overexpression of IQGAP2 did not alter the total protein levels of MEK and ERK but significantly reduced the phosphorylation of both p-MEK and p-ERK (Figures 4A,C–F). In contrast, knockdown of IQGAP2 led to a marked increase in MEK and ERK phosphorylation, indicating enhanced activation of this signaling cascade (Figures 4A,C–F).

We performed additional qPCR assays to detect the mRNA expression of classic downstream proliferation-related target genes in the MEK/ERK MAPK pathway, namely, c-Myc, Cyclin D1 and MMP9, in IQGAP2-OE, shIQGAP2 and control EC9706 cells (Supplementary Figures S3A-C). The results showed that IQGAP2 overexpression markedly reduced the mRNA levels of c-Myc, Cyclin D1 and MMP9 (all *p* < 0.01), consistent with decreased MEK and ERK phosphorylation. Accordingly, IQGAP2 knockdown significantly elevated the expression of these downstream molecules (all *p* < 0.001), which was consistent with hyperactivation of the MEK/ERK signaling cascade.

A rescue experiment using the MEK-specific inhibitor PD98059 was further performed. CCK-8 proliferation assay results showed that PD98059 treatment significantly abolished the promotive effect of IQGAP2 knockdown on ESCC cell proliferation (Supplementary Figure S4). These results clearly demonstrate that the pro-proliferative effect of reduced IQGAP2 in ESCC is dependent on the activation of the MEK/ERK MAPK pathway.

Moreover, we performed all core functional validation experiments in KYSE-150, a widely used moderately differentiated ESCC cell line. We generated IQGAP2-OE and shIQGAP2 KYSE-150 cells and verified transfection efficiency by qPCR (Supplementary Figure S5A). CCK-8 proliferation assay showed completely consistent results with EC9706 cells: overexpression of IQGAP2 significantly inhibited the proliferation of KYSE-150 cells, while knockdown of IQGAP2 markedly promoted cell proliferation (Supplementary Figure S3B). It was also shown that overexpression of IQGAP2 and knockdown of IQGAP2 significantly changes in the target genes of the MEK/ERK MAPK pathway that are directly associated with cell proliferation, including c-Myc, Cyclin D1 and MMP9 (Supplementary Figures S5C-E).

These results demonstrate that our findings are not cell line-specific, and have good generalizability in ESCC cell lines with different differentiation degrees.

## Discussion

IQGAP2, part of the IQGAP family of scaffold proteins, has emerged as a significant tumor suppressor in multiple cancer types. Unlike its homolog IQGAP1, which often promotes tumor progression, IQGAP2 generally inhibits cancer cell proliferation, migration, and invasion, marking it as a promising molecular target for cancer therapy (White et al., 2009; White et al., 2010).

Our findings revealed a significant downregulation of IQGAP2 in ESCC tumor tissues, which is consistent with many reports that identified the downregulation of IQGAP2 across cancers. For example, a consistent finding in cancer research is the downregulation or loss of IQGAP2 expression in tumors. In hepatocellular carcinoma, IQGAP2 expression is frequently reduced, and this reduction correlates with enhanced tumor growth and poor prognosis (White et al., 2009). Functional studies in hepatocellular carcinoma models reveal that IQGAP2 deficiency leads to activation of oncogenic pathways, promoting cell proliferation and metastasis.

Similarly, prostate cancer exhibits decreased IQGAP2 levels, where loss of IQGAP2 is associated with aggressive tumor features (Xie et al., 2012). Restoration of IQGAP2 suppresses prostate cancer cell proliferation and migration, supporting its role as a tumor suppressor. In gastric cancer, reduced IQGAP2 expression correlates with advanced disease and worse survival outcomes (Jin et al., 2008; Xu et al., 2020).

IQGAP2 exerts tumor-suppressive effects through modulation of key signaling pathways. One well-studied mechanism is its inhibition of the Wnt/β-catenin pathway, a critical driver of tumorigenesis. In ovarian cancer, IQGAP2 suppresses Wnt/β-catenin signaling by reducing nuclear β-catenin translocation and transcriptional activity (Deng et al., 2016). This inhibitory effect is crucial in preventing uncontrolled cell proliferation and tumor progression.

IQGAP2 also negatively regulates the MAPK/ERK pathway, which is essential for cell growth and survival in many cancers. It has been demonstrated that overexpression of IQGAP2 inhibits phosphorylation of MEK and ERK, key components of this pathway, thereby suppressing tumor cell proliferation. In bladder cancer, it was shown that reduced IQGAP2 expression promotes epithelial–mesenchymal transition and tumor progression via activation of the MAPK/ERK pathway. Conversely, restoration of IQGAP2 impaired cell growth and lowered ERK phosphorylation levels (Song et al., 2022). Similarly, in breast cancer, Kumar et al. reported that overexpression of IQGAP2 led to a marked decrease in p-MEK and p-ERK, whereas knockdown enhanced ERK phosphorylation (Kumar et al., 2021). This regulation was shown to control cancer cell invasion and survival. These results align with our results in ESCC, where IQGAP2 reduces MEK/ERK activation, indicating a conserved role across tumor types. Together, these studies provide strong evidence that IQGAP2 plays a tumor-suppressive role by inhibiting the MAPK/ERK signaling cascade, making it a compelling candidate for targeted cancer therapies.

Epigenetic silencing of IQGAP2 through promoter methylation has been identified as a mechanism by which cancer cells downregulate its expression. In ovarian cancer, Deng et al. showed that methylation-mediated IQGAP2 silencing leads to activation of Wnt/β-catenin signaling and enhanced tumor growth, highlighting the clinical relevance of restoring IQGAP2 expression (Deng et al., 2016).

In bladder cancer, low IQGAP2 levels are associated with increased invasion and metastasis, supporting its suppressive role in tumor progression (Song et al., 2022). These findings emphasize IQGAP2’s broad tumor suppressor function mediated by common signaling pathways affected in various malignancies.

Clinically, IQGAP2 expression has prognostic significance; low IQGAP2 correlates with poor outcomes and more advanced disease stages in gastric, prostate, and liver cancers (White et al., 2010; Xie et al., 2019; Tang et al., 2021). This positions IQGAP2 as a potential biomarker for cancer prognosis. Therapeutically, targeting the pathways regulated by IQGAP2 or reversing its epigenetic silencing may provide new avenues for cancer treatment. Drugs aiming to demethylate the IQGAP2 promoter or mimicking its function could inhibit oncogenic signaling cascades such as Wnt/β-catenin and MAPK/ERK pathways. Further preclinical and clinical studies are needed to explore these strategies and validate IQGAP2’s utility as a therapeutic target.

Limitations of this study include its reliance on *in vitro* models, which, while informative, do not fully recapitulate the tumor microenvironment or systemic factors influencing ESCC progression. We acknowledge that *in vivo* validation using xenograft models is necessary to further confirm the tumor-suppressive role of IQGAP2. Such studies have not been performed in the present work but represent an important direction for future investigation. Additionally, exploring the prognostic value of IQGAP2 expression in ESCC could aid in patient stratification and personalized therapy development.

## Conclusion

**FIGURE 5:**
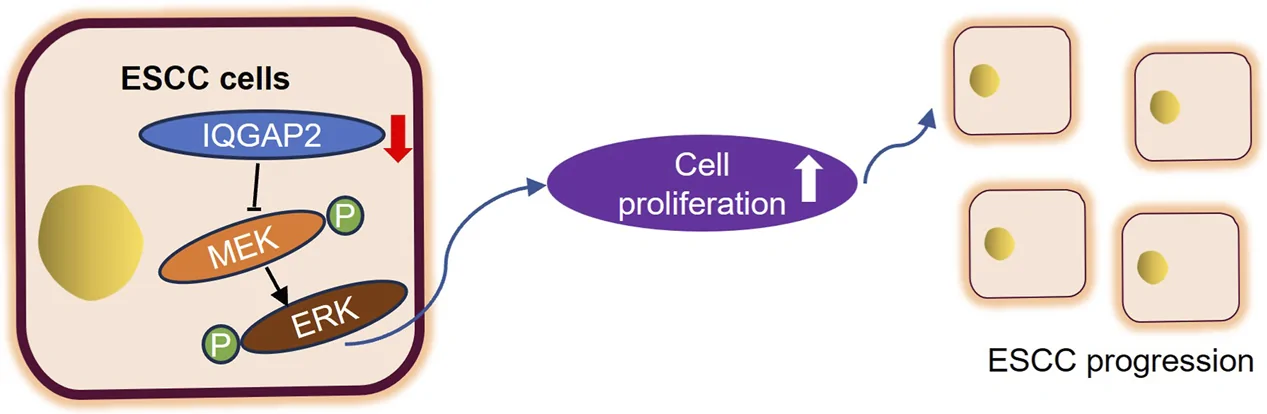
Schematic diagram indicates the proposed role of IQGAP2 in ESCC. Reduced IQGAP2 promotes ESCC cell proliferation via MEK/ERK MAPK pathway.

In summary, this study contributes novel insights into the tumor suppressive functions of IQGAP2 in ESCC through modulation of the MEK/ERK MAPK pathway (Figure 5). Our findings extend the growing body of literature identifying IQGAP2 as a critical regulator of tumorigenesis and provide a rationale for further investigation of IQGAP2 as a therapeutic target in ESCC.

## Data Availability

The original contributions presented in the study are publicly available. This data can be found in the Figshare repository with the DOI 10.6084/m9.figshare.33170783.
