# Supplementary material for: Reduced IQGAP2 promotes esophageal squamous cell carcinoma by regulating MEK/ERK MAPK pathway
Source: Front Genet. 2026 Aug 10;17:1815586. doi: 10.3389/fgene.2026.1815586 (PMC13500107; doi:10.3389/fgene.2026.1815586)
Supplement: Supplementary file 1 [file DataSheet1.docx]

Supplementary materials

Figure S1. Kaplan–Meier analysis shows the correlation between IQGAP2 expression and overall survival of ESCC patients.

Figure S2. Enrichment plot of MAPK pathway (A), GSEA analysis based on patients with low- and high- IQGAP2 expression (B).

Figure S3. Overexpression or knockdown of IQGAP2 in EC9706 cells. The expression levels of c-Myc (A), Cyclin D1 (B) and MMP9 (C) in the EC9706 were measured by RT-qPCR. overexpression: IQGAP2-OE, knockdown: shIQGAP2. Data are represented as mean ± SD. N = 3. * p < 0.05. ** p < 0.01. *** p < 0.001.

Figure S4. MEK-specific inhibitor rescue experiment. CCK-8 assays comparing cell growth of shControl + DMSO, shIQGAP2 + DMSO, shIQGAP2 + PD98059. cells as compared with their control. Data are represented as mean ± SD. N = 3. * and ^#^ p < 0.05. ** and ^##^ p < 0.01. * indicates statistically significant difference between the shIQGAP2 + DMSO group and the shControl + DMSO group; # indicates a statistically significant difference between the shIQGAP2 + DMSO group and the shIQGAP2 + PD98059 group.

Figure S5. Reduced IQGAP2 promotes cell proliferation in KYSE-150 cells. (A) The expression levels of IQGAP2 in the KYSE-150 were measured by RT-qPCR. (B) CCK-8 assays were performed to compare cell proliferation among shIQGAP2, IQGAP2-OE and control cells. The expression levels of c-Myc (C), Cyclin D1 (D) and MMP9 (E) in KYSE-150 were measured by RT-qPCR. Data are represented as mean ± SD. N = 3. * and ^#^ p < 0.05. ** and ^##^ p < 0.01. ***p < 0.001.

**Table S1.** The correlation between IQGAP2 expression and clinicopathological parameters.

| Clinicopathological parameter | Total (n=236) | IQGAP2-high (n=118) | IQGAP2-low (n=118) | P value |
| --- | --- | --- | --- | --- |
| Age (years) |  |  |  | 0.346 |
| ≥64 | 148 | 78 (66.1%) | 70 (59.3%) |  |
| <64 | 88 | 40 (33.9%) | 48 (40.7%) |  |
| Gender |  |  |  | 0.290 |
| Male | 178 | 85 (72.0%) | 93 (78.8%) |  |
| Female | 58 | 33 (28.0%) | 25 (21.2%) |  |
| T stage |  |  |  | 0.014 |
| T1 | 23 | 17 (14.4%) | 6 (5.1%) |  |
| T2 | 53 | 32 (27.1%) | 21 (17.8%) |  |
| T3 | 147 | 63 (53.4%) | 84 (71.2%) |  |
| T4 | 13 | 6 (5.1%) | 7 (5.9%) |  |
| N stage |  |  |  | <0.001 |
| N0 | 153 | 92 (78.0%) | 61 (51.7%) |  |
| N1 | 48 | 18 (15.3%) | 30 (25.4%) |  |
| N2 | 27 | 7 (5.9%) | 20 (16.9%) |  |
| N3 | 8 | 1 (0.8%) | 7 (5.9%) |  |
| M stage |  |  |  | 0.031 |
| M0 | 225 | 116 (98.3%) | 109 (92.4%) |  |
| M1 | 11 | 2 (1.7%) | 9 (7.6%) |  |
| Clinical stage |  |  |  | <0.001 |
| Locoregional (I–II) | 47 | 35 (29.7%) | 12 (10.2%) |  |
| Advanced (III–IV) | 189 | 83 (70.3%) | 106 (89.8%) |  |
